# Ordered Rate Constitutive Theories for Non-Classical Thermofluids Based on Convected Time Derivatives of the Strain and Higher Order Rotation Rate Tensors Using Entropy Inequality

**DOI:** 10.3390/e22040443

**Published:** 2020-04-14

**Authors:** Karan S. Surana, Stephen W. Long

**Affiliations:** Department of Mechanical Engineering, University of Kansas, Lawrence, KS 66048, USA; longs@ku.edu

**Keywords:** ordered rate, constitutive theories, convected time derivatives, strain rate, gradients of rotation rates, representation theorem, integrity

## Abstract

This paper considers non-classical continuum theory for thermoviscous fluids without memory incorporating internal rotation rates resulting from the antisymmetric part of the velocity gradient tensor to derive ordered rate constitutive theories for the Cauchy stress and the Cauchy moment tensor based on entropy inequality and representation theorem. Using the generalization of the conjugate pairs in the entropy inequality, the ordered rate constitutive theory for Cauchy stress tensor considers convected time derivatives of the Green’s strain tensor (or Almansi strain tensor) of up to orders $n_{\varepsilon }$ as its argument tensors and the ordered rate constitutive theory for the Cauchy moment tensor considers convected time derivatives of the symmetric part of the rotation gradient tensor up to orders $n_{\Theta }$. While the convected time derivatives of the strain tensors are well known the convected time derivatives of higher orders of the symmetric part of the rotation gradient tensor need to be derived and are presented in this paper. Complete and general constitutive theories based on integrity using conjugate pairs in the entropy inequality and the generalization of the argument tensors of the constitutive variables and the representation theorem are derived and the material coefficients are established. It is shown that for the type of non-classical thermofluids considered in this paper the dissipation mechanism is an ordered rate mechanism due to convected time derivatives of the strain tensor as well as the convected time derivatives of the symmetric part of the rotation gradient tensor. The derivations of the constitutive theories presented in the paper is basis independent but can be made basis specific depending upon the choice of the specific basis for the constitutive variables and the argument tensors. Simplified linear theories are also presented as subset of the general constitutive theories and are compared with published works.

## 1. Introduction

The conservation and balance laws for non-classical thermofluids incorporating internal rotation rates due to antisymmetric part of the velocity gradient tensor have been presented by Surana et al. [1,2,3,4]. These consist of usual conservation and balance laws of classical continuum mechanics: conservation of mass (CM), balance of linear momenta (BLM), balance of angular momenta (BAM), first and second laws of thermodynamics (FLT, SLT), but require some modification and/or re-derivations and additionally a new balance law, balance of moment of moment (BMM), is needed due to presence of new physics associated with the rotation rates. Yang et al. [5] derived BMM balance law based on static considerations. Surana et al. [6] showed that a balance law must be based on rate considerations and presented a consistent derivation in Lagrangian and Eulerian descriptions. Surana et al. [3] presented an ordered rate constitutive theory for the Cauchy stress tensor using convected time derivatives of up to orders *n* of the Green’s or Almansi strain tensors. However, the constitutive theory for the Cauchy moment tensor was limited to the convected time derivative of order one of the symmetric part of the rotation gradient tensor. In this paper convected time derivatives of the symmetric part of the rotation gradient tensor of up to orders $n_{\Theta }$ are derived. These are then utilized as argument tensors of the Cauchy moment tensor in conjunction with conjugate pairs in entropy inequality. The ordered rate constitutive theories for the Cauchy stress tensor (up to orders $n_{\varepsilon }$) and the Cauchy moment tensor (up to order $n_{\Theta }$) are derived based on theory of isotropic tensors using basis independent approach. The basis independent constitutive theories so derived can be easily made basis specific by basis dependent choices of constitutive variables and their argument tensors. The most general derivations of the constitutive theories for the Cauchy stress and Cauchy moment tensors are based on representation theorem and integrity established using their argument tensors. Constitutive theory for the heat vector is also derived using representation theorem and integrity based on the argument tensors of heat vector. Material coefficients are derived for all ordered rate constitutive theories presented in this paper. Simplified linear ordered rate constitutive theories are presented for the Cauchy stress tensor, Cauchy moment tensor and the heat vector.

The notations used in this paper have been used by the authors in the current literature, nonetheless some description and their use in deriving conservation and balance laws are presented in the following. Over bar is used on quantities to express quantities in the current configuration in Eulerian description, that is, all quantities with over bars are functions of current coordinates $\bar{x}$ and time *t*. The mass density, Helmholtz free-energy density, temperature, and entropy density of the fluid in the current configuration are denoted $\bar{\rho }$, $\bar{\Phi }$, $\bar{\theta }$, and $\bar{\eta }$, respectively, and all are functions of $\bar{x}$. The Cauchy stress tensor in Eulerian description in contravariant basis is denoted by ${\bar{\sigma }}^{\left(0\right)}$. The superscript “0” is used to signify that it is rate of order zero and the lowercase parenthesis distinguish it from the second Piola-Kirchhoff stress tensor $\sigma^{\left[0\right]}$ used in Lagrangian description. Dot on any quantity refers to the material derivative. As explained above undeformed and deformed configurations can be used in the derivatives as long as the final equations from the conservation and balance laws contain ${\bar{x}}_{i}$ and *t* and do not have displacements and strains in them as these are not considered for fluent continua.

### Motivation and New Contributions in the Present Work

In order to illustrate the motivation for this work and the new contribution in this paper for non-classical fluent continua, we must begin with thermoviscous fluids based on classical continuum mechanics (CCM). It is well known that for such fluids the commonly used constitutive theories for deviatoric Cauchy stress tensor ${}_{d}\bar{\sigma }$ is Newton’s law of viscosity. From the CCM point of view this constitutive theory in Equation (1) is a much simplified form of the constitutive theory based on integrity derived using representation theorem in which symmetric part of the velocity gradient tensor and temperature are argument tensors of the deviatoric Cauchy stress tensor in Equation (2):

(1) $$\left[{}_{d}\bar{\sigma }\right]=2\mu \left[\bar{D}\right]+\lambda \mathrm{tr}\left(\left[\bar{D}\right]\right)\left[I\right],$$

(2) $${}_{d}\bar{\sigma }={}_{d}\bar{\sigma }\left(\bar{D},\bar{\theta }\right).$$

We shall show that ${}_{d}\bar{\sigma }$ in this case is basis independent. It is easy to show [7] that $\left[\bar{D}\right]$ is in fact the first convected time derivative $\left[\gamma_{\left(1\right)}\right]$ of the Green’s strain tensor $\left[\varepsilon_{\left[0\right]}\right]$$\left(\mathrm{or}\;\left[\varepsilon \right]\right)$ (a covariant measure). It can also be shown that $\left[\bar{D}\right]$ is the first convected time derivative $\left(\left[\gamma^{\left(1\right)}\right]\right)$ of the Almansi strain tensor $\left[{\bar{\varepsilon }}^{\left[0\right]}\right]$ (a contravariant tensor). Thus,

(3) $$\left[\bar{D}\right]=\left[\gamma_{\left(1\right)}\right]=\left[\gamma^{\left(1\right)}\right].$$

That is, $\left[\bar{D}\right]$ is basis independent, hence ${}_{d}\bar{\sigma }$ in Equation (1) which is entirely dependent on $\left[\bar{D}\right]$ is also basis independent. Constitutive theory in Equation (1) is what is used in fluid mechanics writings. Since $\left[\bar{D}\right]=\left[\gamma_{\left(1\right)}\right]$ and $\left[\bar{D}\right]=\left[\gamma^{\left(1\right)}\right]$, it suggests that one could possibly derive a constitutive theory for deviatoric Cauchy stress tensor using $\left[\gamma_{\left(1\right)}\right]$ as well as $\left[\gamma_{\left(i\right)}\right]\;;\;i=2,3,\ldots ,n$, higher order convected time derivatives of $\left[\varepsilon_{\left[0\right]}\right]$ and θ as its argument tensors. Such stress measure would be contravariant deviatoric Cauchy stress tensor, ${}_{d}{\bar{\sigma }}^{\left(0\right)}$. On the other hand if we decide to choose convected time derivatives $\left[\gamma^{\left(1\right)}\right]$, $\left[\gamma^{\left(i\right)}\right]\;;\;i=2,3,\ldots ,n$, then the stress measure would be covariant, that is, ${}_{d}{\bar{\sigma }}_{\left(0\right)}$. Thus, we can derive constitutive theories for deviatoric Cauchy stress tensor using

(4) $${}_{d}{\bar{\sigma }}^{\left(0\right)}={}_{d}{\bar{\sigma }}^{\left(0\right)}\left(\gamma_{\left(i\right)}\;;\;i=1,2,\ldots ,n,\theta \right)$$

or

(5) $${}_{d}{\bar{\sigma }}_{\left(0\right)}={}_{d}{\bar{\sigma }}_{\left(0\right)}\left(\gamma^{\left(i\right)}\;;\;i=1,2,\ldots ,n,\theta \right).$$

It can be shown that for finite strain rates the constitutive theory resulting from Equation (4) is valid, where as the constitutive theory due to Equation (5) is only valid for small strain rates [7]. Inclusion of $\gamma_{\left(i\right)};\;i=2,3,\ldots ,n$ as argument tensors of deviatoric Cauchy stress ${}_{d}{\bar{\sigma }}^{\left(0\right)}$ makes it basis dependent.

**Remark** **1.** 

*(1)*  *Inclusion of $\left[\gamma_{\left(i\right)}\right];\;i=2,3,\ldots ,n$ as additional argument tensors of deviatoric Cauchy stress ${}_{d}{\bar{\sigma }}^{\left(0\right)}$ may appear non-justifiable based on the conjugate pair $tr\left(\left[\bar{\sigma }\right]\left[\bar{L}\right]\right)=tr\left(\left[\bar{\sigma }\right]\left[\bar{D}\right]\right)$ in the entropy inequality (used in fluid mechanics writings), but if we realize that the rate of work used in the derivation of energy equation only considers velocity and force, then it is not surprising to see the absence of other conjugate terms that would support inclusion of $\left[\gamma_{\left(i\right)}\right];\;i=2,3,\ldots ,n$ as additional argument tensors of ${}_{d}{\bar{\sigma }}^{\left(0\right)}$.*
*(2)*  *From continuum mechanics point of view, we do not seek for applications of the constitutive theory based on $\left[\gamma_{\left(i\right)}\right];\;i=2,3,\ldots ,n$ but rather seek if their inclusion as additional argument tensors of ${}_{d}{\bar{\sigma }}^{\left(0\right)}$ would lead to a consistent constitutive theory which it does.*
*(3)*  *This work described above has been published by Surana et al. in various papers, some are cited in this paper.*
*(4)*  *A clear explanation of the use of higher order convected time derivatives of the strain tensors as argument tensors of deviatoric Cauchy stress for thermoviscous fluids based on CCM and consistency of the resulting constitutive theory and the rationale supporting such derivations is essential in explaining the use of these concepts in non-classical fluent continua incorporating internal rotation rates.*
*(5)*  *We remark that the presence of $\left[\bar{D}\right]$ or $\left[\gamma_{\left(1\right)}\right]$ in the constitutive theory for ${}_{d}{\bar{\sigma }}^{\left(0\right)}$ is responsible for dissipation mechanism in the fluent continua. Thus, each $\left[\gamma_{\left(i\right)}\right];\;i=1,2,\ldots ,n$ contributes to dissipation mechanism, hence the constitutive theory could be referred to as ordered rate constitutive theory for ${}_{d}{\bar{\sigma }}^{\left(0\right)}$.*
*(6)*  *In classical continuum mechanics the constitutive theories for polymeric fluids makes use of convected time derivatives of the strain tensor [8]. The Maxwell model only uses first convected time derivative of Green’s strain tensor, however the Oldroyd-B model uses first as well as second convected time derivatives of the Green’s strain tensor for deriving constitutive theory for the deviatoric Cauchy stress tensor. This suggest that a constitutive theory for non-classical polymeric fluid for Cauchy moment tensor could possibly benefit from the higher order convected rotation rates derived in this paper. This work is currently in progress.*

The non-classical continuum mechanics considered here for thermofluids incorporates antisymmetric part of the velocity gradient tensor (rotation rates) in the conservation and balance laws. This physics is neglected in classical continuum mechanics. The resistance offered to these by the deforming continua results in moments. One could show that upon using Cauchy principle, we can derive the concept of Cauchy moment tensor (contravariant or covariant). The derivation of balance based on this new physics shows that Cauchy moment tensor is symmetric [3] and that Cauchy moment tensor and the symmetric part of the gradients of rotation rate tensor are rate of work conjugate (entropy inequality). The similarity of these conjugate quantities with the conjugate pair $\left[{}_{d}{\bar{\sigma }}^{\left(0\right)}\right]$ and $\left[\bar{D}\right]$ is obvious. Replacing $\left[\bar{D}\right]=\left[\gamma_{\left(1\right)}\right]$ with $\left[\gamma_{\left(i\right)}\right];\;i=1,2,\ldots ,n$ as the argument tensors of ${}_{d}{\bar{\sigma }}^{\left(0\right)}$ suggests, that perhaps we must also consider a parallel and consistent derivation of the constitutive theory for Cauchy moment tensor in which higher order convected time derivatives of rotation rate tensors are considered as its argument tensors. The first concern is that will this lead to a consistent constitutive theory? We show in the paper that it does. The second concern may be, where do we use this theory. We make some comments in the following to address these.

**Comments** 

*(1)*  *Just like ${}_{d}{\bar{\sigma }}^{\left(0\right)}$ and each $\left[\gamma_{\left(i\right)}\right];\;i=1,2,\ldots ,n$ provides a mechanism of dissipation in CCM, Cauchy moment tensor and each convected time derivative of the rotation rate tensor will also result in is additional mechanism of dissipation that is absent in classical mechanics.*
*(2*  *We shall observe that the conjugate pairs in the entropy inequality provide limited information regarding the argument tensors of the constitutive variables. Initial selection of the argument tensors of the constitutive variables based on conjugate pairs in the entropy inequality is augmented in the present work to include additional desired physics based on the higher order convected time derivatives of the Green’s strain tensor for the deviatoric Cauchy stress tensor and higher order convected rotation rates for Cauchy moment tensor. Inclusion of higher order convected time derivatives in both deviatoric Cauchy stress tensor and the Cauchy moment tensor provide hierarchical and ordered rate dissipation mechanism.*
*(3)*  *Do we have applications that require constitutive theory for Cauchy moment tensor with higher order convected time derivatives of the rotation rate tensor? Maybe not at the moment, but the motivation for the present work is to derive more complete and consistent theories for the future that allow more complex physics than what we may be used to at the present. This is the motivation for the work presented here.*
*(4)*  *This paper presents new constitutive theory for non-classical fluent continua, applications are not the thrust of the paper.*

## 2. Preliminaries, Rotation Rates, Gradients of Rotation Rates, Convected Time Derivatives of Strain Tensor and Rotation Gradient Tensor

In the following, a brief explanation of notations is necessary as some of the notations are new. $x_{i}$ and ${\bar{x}}_{i}$ denote the position coordinates of a material point in the reference and current configurations, respectively, in a fixed frame (*x*-frame).

(6) $$\begin{array}{cc} {\bar{x}}_{i} & ={\bar{x}}_{i}\;\left(x_{1},\;x_{2},\;x_{3},\;t\right),\\ x_{i} & =x_{i}\;\left({\bar{x}}_{1},\;{\bar{x}}_{2},\;{\bar{x}}_{3},\;t\right). \end{array}$$

If $\left\{dx\right\}={\left[dx_{1},\;dx_{2},\;dx_{3}\right]}^{T}$ and $\left\{d\bar{x}\right\}={\left[d{\bar{x}}_{1},\;d{\bar{x}}_{2},\;d{\bar{x}}_{3}\right]}^{T}$ are components of length ds and $d\bar{s}$ in reference and current configurations, then

(7) $$\begin{array}{cc} \left\{d{\bar{x}}_{}\right\} & =\left[J\right]\left\{dx_{}\right\};\;\left[J\right]=\left[\frac{\partial \left\{{\bar{x}}_{}\right\}}{\partial \left\{x_{}\right\}}\right],\\ \left\{dx_{}\right\} & =\left[\bar{J}\right]\left\{d{\bar{x}}_{}\right\};\;\left[\bar{J}\right]=\left[\frac{\partial \left\{x_{}\right\}}{\partial \left\{{\bar{x}}_{}\right\}}\right], \end{array}$$

(8) $$\left[\bar{L}\right]=\left[\frac{\partial \left\{{\bar{v}}_{}\right\}}{\partial \left\{{\bar{x}}_{}\right\}}\right];\;{\bar{v}}_{i}={\bar{v}}_{i}\left({\bar{x}}_{1},\;{\bar{x}}_{2},\;{\bar{x}}_{3},\;t\right),$$

(9) $$\begin{array}{cc} \frac{D}{Dt}\left[J\right] & =\left[\bar{L}\right]\left[J\right],\\ \frac{D}{Dt}\left[\bar{J}\right] & =-\left[\bar{J}\right]\left[\bar{L}\right]. \end{array}$$

$\left[J\right]$ and $\left[\bar{J}\right]$ are covariant and contravariant deformation gradient tensors or Jacobian of deformation tensors, and $\left[\bar{L}\right]$ is the velocity gradient tensor. Decomposition of $\left[\bar{L}\right]$ into symmetric tensor $\left[\bar{D}\right]$ and skew symmetric tensor $\left[\bar{W}\right]$ gives

(10) $$\left[\bar{L}\right]=\left[\bar{D}\right]+\left[\bar{W}\right];\;\left[\bar{D}\right]=\frac{1}{2}\left(\left[\bar{L}\right]+{\left[\bar{L}\right]}^{T}\right),\;\left[\bar{W}\right]=\frac{1}{2}\left(\left[\bar{L}\right]-{\left[\bar{L}\right]}^{T}\right).$$

The classical continuum mechanics in Eulerian description only considers $\left[\bar{D}\right]$. $\left[\bar{W}\right]$ is neglected in the conservation and balance laws of CCM in Eulerian description. Thus, the conservation and balance laws and the constitutive theories of CCM in Eulerian description only consider partial physics of the deformation physics contained in $\left[\bar{L}\right]$. $\left[\bar{W}\right]$ contains rotation rates at ${\bar{x}}_{i}$ defined in the following. Consider

(11) $$\bar{\nabla }\times \bar{v}=e_{1}\left(\frac{\partial {\bar{v}}_{3}}{\partial {\bar{x}}_{2}}-\frac{\partial {\bar{v}}_{2}}{\partial {\bar{x}}_{3}}\right)+e_{2}\left(\frac{\partial {\bar{v}}_{1}}{\partial {\bar{x}}_{3}}-\frac{\partial {\bar{v}}_{3}}{\partial {\bar{x}}_{1}}\right)+e_{3}\left(\frac{\partial {\bar{v}}_{2}}{\partial {\bar{x}}_{1}}-\frac{\partial {\bar{v}}_{1}}{\partial {\bar{x}}_{2}}\right),$$

(12) $$\bar{\nabla }\times \bar{v}=e_{1}{\left({\bar{\Theta }}_{x_{1}}\right)}^{\left(1\right)}+e_{2}{\left({\bar{\Theta }}_{x_{2}}\right)}^{\left(1\right)}+e_{3}{\left({\bar{\Theta }}_{x_{3}}\right)}^{\left(1\right)}.$$

Now we can define $\left[\bar{W}\right]$:

(13) $$\left[\bar{W}\right]=\frac{1}{2}\left[\begin{array}{ccc} 0 & -{\left({\bar{\Theta }}_{x_{3}}\right)}^{\left(1\right)} & {\left({\bar{\Theta }}_{x_{2}}\right)}^{\left(1\right)}\\ {\left({\bar{\Theta }}_{x_{3}}\right)}^{\left(1\right)} & 0 & -{\left({\bar{\Theta }}_{x_{1}}\right)}^{\left(1\right)}\\ -{\left({\bar{\Theta }}_{x_{2}}\right)}^{\left(1\right)} & {\left({\bar{\Theta }}_{x_{1}}\right)}^{\left(1\right)} & 0 \end{array}\right].$$

The non-classical continuum theory in this paper considers $\left[\bar{L}\right]$ in its entirety, that is, both $\left[\bar{D}\right]$ and $\left[\bar{W}\right]$ and their convected time derivatives are considered in the derivation of the conservation and balance laws and the constitutive theories.

## 3. Conservation and Balance Laws

The conservation and balance laws for non-classical continuum mechanics in Eulerian description have been presented in References [1,2,3]. As shown by Surana et al. [7], the contravariant Cauchy stress tensor ${\bar{\sigma }}^{\left(0\right)}$ is physical as it is derived using contravariant components of stresses acting on the faces of the deformed tetrahedron in the current configuration. This choice necessitates that we use convected time derivatives of the Green’s strain tensor as rate of work conjugate quantities to ${\bar{\sigma }}^{\left(0\right)}$. On the other hand if we decide to choose covariant Cauchy stress tensor ${\bar{\sigma }}_{\left(0\right)}$ as a stress measure, then the convected time derivatives of Almansi strain tensor must be used as rate of work conjugate quantities to ${\bar{\sigma }}_{\left(0\right)}$. It is well known (see Reference [7]) that the choice of ${\bar{\sigma }}_{\left(0\right)}$ as a stress measure is only meaningful when ${\bar{x}}_{i}≅x_{i}$. This precludes its use for finite strain rate processes. Jaumann stress measure ${}^{\left(0\right)}{\bar{\sigma }}^{J}$ that is average of ${\bar{\sigma }}^{\left(0\right)}$ and ${\bar{\sigma }}_{\left(0\right)}$ obviously suffers from the same limitations as ${\bar{\sigma }}_{\left(0\right)}$. We shall show in a later section that we have the following conjugate pairs:

(14) $$\left.\begin{array}{c} \left[{\bar{\sigma }}^{\left(0\right)}\right],\left[\gamma_{\left(i\right)}\right]\\ \left[{\bar{\sigma }}_{\left(0\right)}\right],\left[\gamma^{\left(i\right)}\right]\\ \left[{}^{\left(0\right)}{\bar{\sigma }}^{J}\right],\left[{}^{\left(i\right)}\gamma^{J}\right] \end{array}\right\}i=1,2,\ldots ,n,$$

in which $\left[\gamma_{\left(i\right)}\right]$, $\left[\gamma^{\left(i\right)}\right]$ are convected time derivatives of the Green’s and Almansi strain tensors and $\left[{}^{\left(i\right)}\gamma^{J}\right]$ are Jaumann rates. These conjugate pairs are clearly basis dependent. That is, if we choose a stress tensor in a preferred basis then the basis for the strain measure and its convected time derivatives is automatically established based on conjugate pair(s). Instead of choosing conjugate pairs from Equation (14) we consider the basis independent measures in the derivation of the constitutive theories. Let the following be conjugate pair(s):

(15) $$\left[{}^{\left(0\right)}\bar{\sigma }\right],\left[{}^{\left(i\right)}\gamma \right];\;i=1,2,\ldots ,n_{\varepsilon }.$$

Similarly, for the Cauchy moment tensor we consider similar basis independent conjugate pairs in the derivation of the constitutive theory for the Cauchy moment tensor:

(16) 0m¯,iγiΘ;i=1,2,…,nθ

in which iγiΘ;i=1,2,…,nθ are the convected time derivatives of the rotation gradient tensor. The derivations of these is presented in a following section. Choosing desired pairs from Equation (14) and using them in Equation (15) will yield the constitutive theory for Cauchy stress tensor in the desired basis. The same will hold for Equation (16). We summarize the conservation and balance laws for non-classical continuum mechanics (NCCM) based on internal rotation rates [1,2,3] in Eulerian description in the following. These consist of conservation of mass (CM), balance of linear momenta (BLM), balance of angular momenta (BAM), balance of moment of moments (BMM), first law of thermodynamics (FLT), and the second law of thermodynamics (SLT):

(17) $$\begin{array}{cc} \; & \frac{\partial \bar{\rho }}{\partial t}+\frac{\partial }{\partial {\bar{x}}_{i}}\left(\bar{\rho }{\bar{v}}_{i}\right)=0\;\left(\mathrm{CM}\right), \end{array}$$

(18) $$\begin{array}{cc} \; & \bar{\rho }\frac{\partial {\bar{v}}_{i}}{\partial t}+\bar{\rho }{\bar{v}}_{j}\frac{\partial {\bar{v}}_{i}}{\partial {\bar{x}}_{j}}-\bar{\rho }{\bar{F}}_{i}^{b}-\frac{\partial }{\partial {\bar{x}}_{j}}\left({}^{\left(0\right)}{\bar{\sigma }}_{ji}\right)=0\;\left(\mathrm{BLM}\right), \end{array}$$

(19) $$\begin{array}{cc} \; & {}^{\left(0\right)}{\bar{m}}_{pk,p}+\epsilon_{ijk}{}^{\left(0\right)}{\bar{\sigma }}_{ij}=0\;\left(\mathrm{BAM}\right), \end{array}$$

(20) $$\begin{array}{cc} \; & \epsilon_{ijk}{}^{\left(0\right)}{\bar{m}}_{ij}=0\;\left(\mathrm{BMM}\right), \end{array}$$

(21) ρ¯De¯Dt+∂q¯i∂x¯i−0σ¯ji∂v¯i∂x¯j−0m¯ji1γiΘij−1Θ¯·ϵ:0σ¯=0FLT,

(22) ρ¯Dϕ¯Dt+η¯Dθ¯Dt+q¯ig¯iθ¯−0σ¯ji∂v¯i∂x¯j−0m¯ji1γiΘij−1Θ¯·ϵ:0σ¯≤0SLT,

in which e¯,η¯,θ¯,q¯,g¯,1γiΘ,and1Θ¯ are specific internal energy, entropy density, absolute temperature, heat flux, temperature gradients, gradients of the first convected internal rotation rates, and internal rotation rates, respectively.

**Remark** **2.** 

*(1)*  *Equations (17)–(21) are a system of eight partial differential equations (CM (1), BLM (3), BAM (3), FLT (1)) in twenty three dependent variables ($\bar{\rho }$ (1), $\bar{v}$ (3), ${}^{\left(0\right)}\bar{\sigma }$ (9), $\bar{q}$ (3), $\bar{\theta }$ (1), ${}^{\left(0\right)}\bar{m}$ (6)), thus we need fifteen equations for the mathematical model defined by Equations (17)–(21) to have closure. These are obtained from the constitutive theory. We note that $\bar{\phi },\;\bar{e},\;and,\;\bar{\eta }$ are not dependent variables as these can be expressed in terms of the variables already considered.*
*(2)*  *While in fluid mechanics and gas dynamics the entropy inequality seems to play a very limited role based on the writing on the subject, primarily in inferring whether a process is irreversible or not or whether the calculated solutions violate the SLT, which could happen if the calculated solutions are in error. In view of the fact that all fluids must possess viscosity, irreversibility in fluid mechanics and gas dynamics is intrinsic hence is a given fact. Thus, it appears that the role of entropy inequality is rather peripheral. In continuum mechanics (classical or non-classical) the role of entropy inequality is rather central in the development of the constitutive theories:*
  *(i)*  *determination of constitutive variables and the initial determination of their argument tensors based on the conjugate pairs in one of the most significant and crucial conclusions we are able to draw from the entropy inequality. This gives us a starting point in the derivation of the constitutive theories.*
  *(ii)*  *Helmholtz free energy density in the entropy inequality facilitates constitutive theories for equilibrium stress or total stress for elastic solid continua depending upon the physics considered.*
  *(iii)*  *The role of Helmholtz free energy density and the rate of work conjugate pairs in entropy inequality are vital in the derivation of the constitutive theories for solid continua as well as fluent continua.*
*(3)*  *We remark that once the constitutive theories are derived using the conjugate pairs in the entropy inequality, the entropy inequality condition is identically satisfied by the solution obtained using the balance laws incorporating these constitutive theories. When using numerical methods such as finite element method $L_{2}$-norm of the residuals over the discretization [9,10] can be used to ensure that the calculated solution is indeed the solution of the mathematical model consisting of the conservation and balance laws incorporating constitutive theories derived using entropy inequality. Hence, every calculated solution with sufficient accuracy always satisfies SLT provided the conditions resulting from the conjugate pairs are not violated in the derivation of the constitutive theories.*
*(4)*  *Thus, in continuum mechanics the entropy inequality plays a central role as without it thermodynamically constitutive theories are not possible. FLT and SLT need to be recast in slightly different forms so that the conjugate pairs helpful in deriving constitutive theories can be established.*

## 4. Constitutive Theories

First we consider some preliminary material to present alternate forms of some terms in the FLT and the SLT. Let 1γiΘ represent the gradient of the rotation rate tensor (basis independent), then

(23) 1γiΘ=∂1Θ¯∂1x¯.

We note that 1γiΘ is also the first convected time derivative of the rotation gradient tensor. We consider decomposition of $\left[{}^{\left(0\right)}\bar{\sigma }\right]$, $\left[{}^{\left(0\right)}\bar{m}\right]$, $\left[\bar{L}\right]$, and 1γiΘ into symmetric and antisymmetric tensors (indicated by back subscripts *s* and *a*):

(24) 0σ¯=(s)0σ¯+(a)0σ¯0m¯=(s)0m¯+(a)0m¯1γiΘ=(s)1γiΘ+(a)1γiΘ.

We note the following:

(25) 0σ¯ji∂v¯i∂x¯j=(s)0σ¯ji+(s)0σ¯jiD¯ij+W¯ij=(s)0σ¯jiD¯ij+(s)0σ¯jiW¯ij.

We can also write Equation (25) as

(26) tr0σ¯L¯=tr(s)0σ¯D¯+tr(a)0σ¯W¯.

Similarly,

(27) 0m¯ji1γiΘij=(s)0m¯ji+(a)0m¯ji(s)1γiΘij+(a)1γiΘij=(s)0m¯ji(s)1γiΘij+(a)0m¯ji(a)1γiΘij

(28) =(s)0m¯ji(s)1γiΘijas0m¯issymmetric.

We can also write Equation (28) as

(29) tr0m¯1γiΘ=tr(s)0m¯(s)1γiΘ.

With simple calculations we can show that

(30) tr(a)0m¯W¯=−1Θ¯·ϵ:0σ¯.

Using Equations (26), (29), and (30) in FLT and SLT (Equations (21) and (22)) we can obtain

(31) ρ¯De¯Dt+∂q¯i∂x¯i−(s)0σ¯jiD¯ij−(s)0m¯ji(s)1γiΘij=0,

(32) ρ¯Dϕ¯Dt+η¯Dθ¯Dt+q¯ig¯iθ¯−(s)0σ¯jiD¯ij−(s)0m¯ji(s)1γiΘij≤0.

Equations (17)–(20) and (31), (32) constitute the complete mathematical model in Eulerian description.

### 4.1. Constitutive Variables and Their Argument Tensors

From Equations (17)–(20) and (31), (32), we determine that $\bar{\phi }$, $\bar{\eta }$, (s)0σ¯, (s)0m¯, and $\bar{q}$ are constitutive variables. At this stage the argument tensors of (s)0σ¯, (s)0m¯, and $\bar{q}$ can be established from the rate of work conjugate pairs (s)0σ¯jiD¯ij, (s)0m¯ji(s)1γiΘij, and the conjugate pair $\frac{{\bar{q}}_{i}{\bar{g}}_{i}}{\bar{\theta }}$. From conservation of mass in Lagrangian description $\rho_{0}=\left|J\right|\rho$, that is, compressibility is due to $\left|J\right|=\frac{\rho_{0}}{\rho }$, hence it is fitting to consider $\frac{1}{\bar{\rho }}$ as an argument tensor in Eulerian description as opposed to $\bar{\rho }$ of the constitutive variables. At a later stage dependence on $\frac{1}{\bar{\rho }}$ can be replaced by dependence on $\bar{\rho }$ (using calculus). Furthermore we consider $\bar{\theta }$ as argument tensor of the constitutive variables (thermoviscous fluid). Thus, we can write

(33) (s)0σ¯=(s)0σ¯1ρ¯,D¯,θ¯,

(34) (s)0m¯=(s)0m¯1ρ¯,(s)1γiΘ,θ¯,

(35) $$\begin{array}{c} \bar{q}=\bar{q}\;\left(\frac{1}{\bar{\rho }},\;\bar{g},\;\bar{\theta }\right). \end{array}$$

Argument tensors of $\bar{\phi }$ and $\bar{\eta }$ can be established using principle of equipresence [7,11]

(36) ϕ¯=ϕ¯1ρ¯,D¯,(s)1γiΘ,g¯,θ¯,

(37) η¯=η¯1ρ¯,D¯,(s)1γiΘ,g¯,θ¯.

However, we have not used principle of equipresence for ${}^{\left(0\right)}\bar{\sigma }$, ${}^{\left(0\right)}\bar{m}$, and $\bar{q}$ as the conjugate pairs specifically dictate their argument tensors as used in Equations (33)–(35).

### 4.2. Convected Time Derivatives of Strain Tensors and Convected Rotation Rates

The argument tensors of the constitutive variables in Equations (33)–(37) can be enhanced to permit more comprehensive constitutive theories. Convected time derivatives of the Green’s strain tensor and the Almansi strain tensor have been presented in Reference [7]. We summarize these in the following.

#### 4.2.1. Convected Time Derivatives of the Green’s Strain Tensor

If $\left[\varepsilon_{\left[0\right]}\right]$ is Green’s strain tensor, then

(38) $$\begin{array}{c} \left[\gamma_{\left[1\right]}\right]=\frac{D}{Dt}\left[\varepsilon_{\left[0\right]}\right]={\left[J\right]}^{T}\left[\gamma_{\left(1\right)}\right]\left[J\right], \end{array}$$

(39) $$\left.\begin{array}{cc} \; & \frac{D}{Dt}\left[\gamma_{\left[k-1\right]}\right]=\left[\gamma_{\left[k\right]}\right]\\ \; & \left[\gamma_{\left[k\right]}\right]={\left[J\right]}^{T}\left[\gamma_{\left(k\right)}\right]\left[J\right]\\ \; & \left[\gamma_{\left(k\right)}\right]=\frac{D}{Dt}\left[\gamma_{\left(k-1\right)}\right]+{\left[\bar{L}\right]}^{T}\left[\gamma_{\left(k-1\right)}\right]+\left[\gamma_{\left(k-1\right)}\right]\left[\bar{L}\right] \end{array}\right\}k=2,3,\ldots ,n_{\varepsilon },$$

in which $\left[\gamma_{\left(j\right)}\right]\;;\;j=1,2,\ldots ,n_{\varepsilon }$ are the convected time derivatives of the Green’s strain tensor $\left[\varepsilon_{\left[0\right]}\right]$ of orders $1,2,\ldots ,n_{\varepsilon }$. We can show that

(40) $$\left[\gamma_{\left(1\right)}\right]=\left[\bar{D}\right]=\frac{1}{2}\left(\left[\bar{L}\right]+{\left[\bar{L}\right]}^{T}\right).$$

These are obviously covariant measures.

#### 4.2.2. Convected Time Derivatives of Almansi Strain Tensor

Let $\left[{\bar{\varepsilon }}^{\left[0\right]}\right]$ be Almansi strain tensor, then we have the following

(41) $$\left[\gamma^{\left[1\right]}\right]=\frac{D}{Dt}\left[{\bar{\varepsilon }}^{\left[0\right]}\right]=\left[\bar{J}\right]\left[\gamma^{\left(1\right)}\right]{\left[\bar{J}\right]}^{T},$$

(42) $$\left.\begin{array}{cc} \; & \left[\gamma^{\left[k\right]}\right]=\frac{D}{Dt}\left[\gamma^{\left[k-1\right]}\right]\\ \; & \left[\gamma^{\left[k\right]}\right]=\left[\bar{J}\right]\left[\gamma^{\left(k\right)}\right]{\left[\bar{J}\right]}^{T}\\ \; & \left[\gamma^{\left(k\right)}\right]=\frac{D}{Dt}\left[\gamma^{\left(k-1\right)}\right]-\left[\bar{L}\right]\left[\gamma^{\left(k-1\right)}\right]-\left[\gamma^{\left(k-1\right)}\right]{\left[\bar{L}\right]}^{T} \end{array}\right\}k=2,3,\ldots ,n_{\varepsilon },$$

in which $\left[\gamma^{\left(j\right)}\right]\;;\;j=1,2,\ldots ,n_{\varepsilon }$ are the convected time derivatives of orders $1,2,\ldots ,n_{\varepsilon }$ of the Almansi strain tensor $\left[{\bar{\varepsilon }}^{\left[0\right]}\right]$. We can also show that

(43) $$\left[\gamma^{\left(1\right)}\right]=\left[\bar{D}\right]=\frac{1}{2}\left(\left[\bar{L}\right]+{\left[\bar{L}\right]}^{T}\right).$$

**Remark** **3.** 

*(1)*  *We note that*
  (44) $$\left[\gamma_{\left(1\right)}\right]=\left[\gamma^{\left(1\right)}\right]=\left[\bar{D}\right].$$
*Thus the first convected time derivatives of $\left[\varepsilon_{\left[0\right]}\right]$ and $\left[{\bar{\varepsilon }}^{\left[0\right]}\right]$ are basis independent.*
*(2)*  *Jaumann rates $\left[{}^{\left(k\right)}\gamma^{J}\right]$ are the average of $\left[\gamma_{\left(k\right)}\right]$ and $\left[\gamma^{\left(k\right)}\right]$ rates, that is,*
  (45) $$\left[{}^{\left(k\right)}\gamma^{J}\right]=\frac{1}{2}\left(\left[\gamma_{\left(k\right)}\right]+\left[\gamma^{\left(k\right)}\right]\right)\;;\;k=1,2,\ldots ,n_{\varepsilon }.$$

#### 4.2.3. Gradients of the Higher Order Convected Rotation Rates

To our knowledge, the gradients of the higher order convected rotation rates presented in the following is its first presentation. Consider

(46) $$\nabla \times v=e_{1}{\left(\Theta_{x_{1}}\right)}_{\left(1\right)}+e_{2}{\left(\Theta_{x_{2}}\right)}_{\left(1\right)}+e_{2}{\left(\Theta_{x_{3}}\right)}_{\left(1\right)},$$

and

(47) $$\bar{\nabla }\times \bar{v}=e_{1}{\left({\bar{\Theta }}_{x_{1}}\right)}^{\left(1\right)}+e_{2}{\left({\bar{\Theta }}_{x_{2}}\right)}^{\left(1\right)}+e_{2}{\left({\bar{\Theta }}_{x_{3}}\right)}^{\left(1\right)}.$$

Thus, we have

(48) γiΘ1=∂Θ1∂x,

(49) γiΘ1=∂Θ¯1∂x¯,

in which γiΘ1 and γiΘ1 are gradients of the rotation rates (of order one) in co- and contra-variant bases. The symmetric and the antisymmetric components of γiΘ1 and γiΘ1 are given by

(50) sγiΘ1=12γiΘ1+γiΘ1T,aγiΘ1=12γiΘ1−γiΘ1T

and

(51) sγiΘ1=12γiΘ1+γiΘ1T,aγiΘ1=12γiΘ1−γiΘ1T.

We can define the second Piola-Kirchhoff tensors corresponding to sγiΘ1 and sγiΘ1 by

(52) sγiΘ1=JsγiΘ1JTandsγiΘ1=J¯sγiΘ1J¯T.

Following the details in Reference [7] and Section 4.2.1 and Section 4.2.2 we can write the following for sγiΘk;k=2,3,…,nΘ, the symmetric parts of the gradient of the higher order convected rotation rates in covariant basis

(53) sγiΘk=DDtsγiΘk−1sγiΘk=JsγiΘkJTsγiΘk=DDtsγiΘk−1+L¯TsγiΘk−1+sγiΘk−1L¯k=2,3,…,nΘ.

Likewise, for sγiΘk;k=2,3,…,nΘ, the symmetric parts of the gradient of the higher order convected rotation rates in contravariant basis can be defined by

(54) sγiΘk=DDtsγiΘk−1sγiΘk=J¯sγiΘkJ¯TsγiΘk=DDtsγiΘk−1−L¯sγiΘk−1−sγiΘk−1L¯Tk=2,3,…,nΘ.

Symmetric parts of the gradient of the convected rotation rate tensors called Jaumann tensors can be defined as

(55) ksγiΘiJ=12sγiΘk+sγiΘk;k=1,2,…,nΘ.

In order to make the derivation of the constitutive theories basis independent we use the notation (s)kγiΘ;k=1,2,…,nΘ for the symmetric parts of the gradient of convected rotation rates. Depending upon the choice of the basis for the Cauchy moment tensor, that is, contra- or co-variant or Jaumann basis we can choose the corresponding conjugates as sγiΘk, sγiΘk, and ksγiΘiJ, respectively.

### 4.3. Final Choice of Argument Tensors for the Constitutive Variables

Recall the constitutive variables and their argument tensors defined by Equations (33)–(37). We can generalize this choice of the argument tensors of Cauchy stress and moment tensors by replacing

(56) D¯i.e.,γ1orγ1or1γJbyiγ;k=1,2,…,nε,(s)1γiΘby(s)kγiΘ;k=1,2,…,nΘ.

Thus, now we have

(57) (s)0σ¯=(s)0σ¯1ρ¯,kγ,θ¯;k=1,2,…,nε,

(58) (s)0m¯=(s)0m¯1ρ¯,(s)kγiΘ,θ¯;k=1,2,…,nΘ,

(59) $$\begin{array}{c} \bar{q}=\bar{q}\;\left(\frac{1}{\bar{\rho }},\;\bar{g},\;\bar{\theta }\right), \end{array}$$

(60) ϕ¯=ϕ¯1ρ¯,iγ,(s)jγiΘ,g¯,θ¯;i=1,2,…,nεj=1,2,…,nΘ.

### 4.4. Entropy Inequality and Constitutive Theories

Consider entropy inequality Equation (32) and the argument tensors of $\bar{\phi }$ in Equation (60). Using the chain rule $\overset{\;.}{\bar{\phi }}=\frac{D\bar{\phi }}{Dt}$ can now be obtained:

(61) ϕ¯.=Dϕ¯Dt=∂ϕ¯∂1ρ¯−1ρ¯2ρ¯.+∑i=1nε∂ϕ¯∂iγilkiγ.ilk+∑j=1nΘ∂ϕ¯∂(s)jγiΘlk(s)jγ.iΘlk+∂ϕ¯∂g¯ig¯.i+∂ϕ¯∂θ¯θ¯..

From the continuity equation

(62) Dρ¯Dt=ρ¯.=ρ¯∇¯·v¯=−ρ¯D¯kk=−ρ¯1γikk=−ρ¯1γilkδlk.

Using Equation (62) in Equation (61)

(63) ϕ¯.=1ρ¯∂ϕ¯∂1ρ¯1γilkδlk+∑i=1nε∂ϕ¯∂iγilkiγ.ilk+∑j=1nΘ∂ϕ¯∂(s)jγiΘlk(s)jγ.iΘlk+∂ϕ¯∂g¯ig¯.i+∂ϕ¯∂θ¯θ¯..

We note that

(64) $$\frac{\partial \bar{\phi }}{\partial \left(\frac{1}{\bar{\rho }}\right)}={-\bar{\rho }}^{2}\frac{\partial \bar{\phi }}{\partial \bar{\rho }}.$$

Using Equation (64) in Equation (63)

(65) ϕ¯.=−ρ¯∂ϕ¯∂ρ¯1γilkδlk+∑i=1nε∂ϕ¯∂iγilkiγ.ilk+∑j=1nΘ∂ϕ¯∂(s)jγiΘlk(s)jγ.iΘlk+∂ϕ¯∂g¯ig¯.i+∂ϕ¯∂θ¯θ¯..

Substituting from Equation (65) in entropy inequality Equation (32) we obtain

(66) ρ¯−ρ¯∂ϕ¯∂ρ¯1γilkδlk+∑i=1nε∂ϕ¯∂iγilkiγ.ilk+∑j=1nΘ∂ϕ¯∂(s)jγiΘlk(s)jγ.iΘlk+∂ϕ¯∂g¯ig¯.i+∂ϕ¯∂θ¯θ¯.+η¯Dθ¯Dt+q¯ig¯iθ¯−(s)0σ¯lk1γilk−(s)0m¯lk(s)1γiΘlk≤0.

Regrouping terms in Equation (66) we can write

(67) −ρ¯2∂ϕ¯∂ρ¯δlk−(s)0σ¯lk1γilk+ρ¯∑i=1nε∂ϕ¯∂iγilkiγ.ilk+ρ¯∑j=1nΘ∂ϕ¯∂(s)jγiΘlk(s)jγ.iΘlk+ρ¯∂ϕ¯∂θ¯+η¯θ¯.+ρ¯∂ϕ¯∂g¯ig¯.i+q¯ig¯iθ¯−(s)0m¯lk(s)1γiΘlk≤0.

For Equation (67) to hold for arbitrary but admissible choices of $\left[{}^{\left(i\right)}\gamma \right]\;;\;i=1,2,\ldots ,n_{\varepsilon }$, (s)jγiΘ;j=1,2,…,nΘ, $\overset{\;.}{\bar{g}}$, and $\overset{\;.}{\bar{\theta }}$, the following must hold:

(68) ρ¯∂ϕ¯∂iγilk=0⇒ϕ¯≠ϕ¯iγ;i=1,2,…,nε,

(69) ρ¯∂ϕ¯∂(s)jγiΘlk=0⇒ϕ¯≠ϕ¯(s)jγiΘ;j=1,2,…,nΘ,

(70) $$\begin{array}{c} \bar{\rho }\frac{\partial \bar{\phi }}{\partial {\bar{g}}_{i}}=0\Rightarrow \bar{\phi }\neq \bar{\phi }\;\left(\bar{g}\right), \end{array}$$

(71) $$\begin{array}{c} \bar{\rho }\left(\frac{\partial \bar{\phi }}{\partial \bar{\theta }}+\bar{\eta }\right)=0\Rightarrow \bar{\eta }=-\frac{\partial \bar{\phi }}{\partial \bar{\theta }}. \end{array}$$

The entropy inequality Equation (67) reduces to

(72) −ρ¯2∂ϕ¯∂ρ¯δlk−(s)0σ¯lk1γilk+q¯ig¯iθ¯−(s)0m¯lk(s)1γiΘlk≤0.

Equations (68)–(71) are fundamental relations resulting from the entropy inequality and Equation (72) is the reduced form of the entropy inequality.

**Remark** **4.** 

*(1)*  *Equation (68) implies that $\bar{\phi }$ is not a function of $\left[{}^{\left(i\right)}\gamma \right]\;;\;i=1,2,\ldots ,n_{\varepsilon }$.*
*(2)*  *Equation (69) shows that $\bar{\phi }$ is not a function of (s)jγiΘ;j=1,2,…,nΘ.*
*(3)*  *From Equation (70) we can conclude that $\bar{g}$ is not an argument tensor of $\bar{\phi }$.*
*(4)*  *Equation (71) allows us to determine $\bar{\eta }$ using $\bar{\phi }$, hence $\bar{\eta }$ is not a constitutive variable.*
*(5)*  *From remarks (1)–(4) we have that $\bar{\phi }=\bar{\phi }\left(\bar{\rho },\;\bar{\theta }\right)$.*
*(6)*  *The entropy inequality Equation (72) is essential in the form stated in Equation (72). Setting coefficient of 1γilk in the first term of Equation (72) to zero would imply that*
  (73) (s)0σ¯lk=−ρ¯2∂ϕ¯∂ρ¯δlk
*which implies that (s)0σ¯ is not a function of $\left[{}^{\left(i\right)}\gamma \right]\;;\;i=1,2,\ldots ,n_{\varepsilon }$, which is contrary to Equation (57). The entropy inequality Equation (72) in this form is unable to provide further details regarding the derivations of the constitutive theories (specifically for (s)0σ¯).*

In view of the remarks, the choice of constitutive variables and their argument tensors can be modified:

(74) $$\begin{array}{c} \bar{\phi }=\bar{\phi }\;\left(\bar{\rho },\;\bar{\theta }\right), \end{array}$$

(75) (s)0σ¯=(s)0σ¯ρ¯,iγ,θ¯;i=1,2,…,nε,

(76) (s)0m¯=(s)0m¯ρ¯,(s)jγiΘ,θ¯;j=1,2,…,nΘ,

(77) $$\begin{array}{c} \bar{q}=\bar{q}\;\left(\bar{\rho },\;\bar{g},\;\bar{\theta }\right). \end{array}$$

### 4.5. Decomposition of Cauchy Stress Tensor (s)0σ¯

We decompose Cauchy stress tensor (s)0σ¯ into equilibrium Cauchy stress tensor, e(s)0σ¯, and deviatoric stress tensor, d(s)0σ¯, that is,

(78) (s)0σ¯=e(s)0σ¯+d(s)0σ¯

in which we consider the following

(79) e(s)0σ¯=e(s)0σ¯ρ¯,θ¯,

(80) d(s)0σ¯=d(s)0σ¯ρ¯,iγ,θ¯;i=1,2,…,nε,

(81) andd(s)0σ¯ρ¯,θ¯=0.

That is, e(s)0σ¯ is not a function of ${}^{\left(i\right)}\gamma ;\;i=1,2,\ldots ,n_{\varepsilon }$ and d(s)0σ¯ vanishes when ${}^{\left(i\right)}\gamma ;\;i=1,2,\ldots ,n_{\varepsilon }$ are zero. Substituting Equation (78) in Equation (72)

(82) −ρ¯2∂ϕ¯∂ρ¯δlk−e(s)0σ¯lk−d(s)0σ¯lk1γilk+q¯ig¯iθ¯−(s)0m¯lk(s)1γiΘlk≤0,

or

(83) −ρ¯2∂ϕ¯∂ρ¯δlk−e(s)0σ¯lk1γilk−d(s)0σ¯lk1γilk+q¯ig¯iθ¯−(s)0m¯lk(s)1γiΘlk≤0.

### 4.6. Constitutive Theory for Equilibrium Stress e(s)0σ¯

#### 4.6.1. Compressible Matter

We note that e(s)0σ¯=e(s)0σ¯ρ¯,θ¯ and $\bar{\phi }=\bar{\phi }\;\left(\bar{\rho },\;\bar{\theta }\right)$. Thus, we can derive constitutive theory for e(s)0σ¯=e(s)0σ¯ρ¯,θ¯ by setting coefficient of 1γilk in the first term of Equation (83) to zero, that is,

(84) e(s)0σ¯lk=−ρ¯2∂ϕ¯∂ρ¯δlk=p¯ρ¯,θ¯δlk,ore(s)0σ¯=p¯ρ¯,θ¯I,

in which

(85) $$\bar{p}\;\left(\bar{\rho },\;\bar{\theta }\right)=-{\bar{\rho }}^{2}\frac{\partial \bar{\phi }}{\partial \bar{\rho }}.$$

$\bar{p}\;\left(\bar{\rho },\;\bar{\theta }\right)$ is called thermodynamic pressure. If we consider compressive pressure $\bar{p}\;\left(\bar{\rho },\;\bar{\theta }\right)$ to be positive, then $\bar{p}\;\left(\bar{\rho },\;\bar{\theta }\right)$ in Equation (84) can be replaced by $-\bar{p}\;\left(\bar{\rho },\;\bar{\theta }\right)$. Using Equation (84), the entropy inequality reduces to

(86) −d(s)0σ¯lk1γilk−(s)0m¯lk(s)1γiΘlk+q¯ig¯iθ¯≤0.

#### 4.6.2. Incompressible Matter

For incompressible matter density is constant, hence $\bar{\rho }=\rho_{0}$, $\frac{\partial \bar{\phi }}{\partial \bar{\rho }}=0$, and e(s)0σ¯=e(s)0σ¯θ¯, thus the constitutive theory for e(s)0σ¯ for incompressible matter cannot be derived using $\frac{\partial \bar{\phi }}{\partial \bar{\rho }}$, that is, using Equation (84). The problem arises due to the fact that the incompressibility condition

(87) ∇¯·v¯=trD¯=tr1γ=1γilkδlk=0

is not present in the entropy inequality, but can be incorporated in it by simply adding the following to the entropy inequality Equation (83)

(88) p¯θ¯1γilkδlk=0,

in which $\bar{p}\;\left(\bar{\theta }\right)$ is an arbitrary Lagrange multiplier. This yields:

(89) −ρ¯2∂ϕ¯∂ρ¯δlk−e(s)0σ¯lk1γilk+p¯θ¯1γilkδlk−d(s)0σ¯lk1γilk−(s)0m¯lk(s)1γiΘlk+q¯ig¯iθ¯≤0.

Using $\frac{\partial \bar{\phi }}{\partial \bar{\rho }}=0$ and regrouping terms in Equation (89),

(90) p¯θ¯δlk−e(s)0σ¯lk1γilk+q¯ig¯iθ¯−d(s)0σ¯lk1γilk−(s)0m¯lk(s)1γiΘlk≤0.

Since e(s)0σ¯=e(s)0σ¯θ¯, we can derive constitutive theory for e(s)0σ¯ by setting the coefficient of 1γilk in Equation (90) to zero, that is,

(91) e(s)0σ¯lk=p¯θ¯δlk,or(s)0σ¯=p¯θ¯I.

$\bar{p}\;\left(\bar{\theta }\right)$ is called mechanical pressure. Since $\bar{p}\;\left(\bar{\theta }\right)$ is an arbitrary Lagrange multiplier, $\bar{p}\;\left(\bar{\theta }\right)$ cannot be determined from the deformation field. The entropy inequality reduces to the same as in the compressible case, that is, Equation (86).

### 4.7. Constitutive Theories for d(s)0σ¯, (s)0m¯, and $\bar{q}$

The constitutive variables and their argument tensors are now given in the following.

(92) d(s)0σ¯=d(s)0σ¯ρ¯,iγ,θ¯;i=1,2,…,nε,

(93) (s)0m¯=(s)0m¯ρ¯,(s)jγiΘ,θ¯;j=1,2,…,nΘ,

(94) $$\begin{array}{cc} \bar{\phi } & =\bar{\phi }\;\left(\bar{\theta }\right), \end{array}$$

and

(95) e(s)0σ¯=p¯ρ¯,θ¯I;Compressiblee(s)0σ¯=p¯θ¯I;Incompressible,

(96) $$\bar{q}=\bar{q}\;\left(\bar{\rho },\;\bar{g},\;\bar{\theta }\right).$$

The final form of the entropy inequality is given by

(97) q¯ig¯iθ¯−d(s)0σ¯lk1γilk−(s)0m¯lk(s)1γiΘlk≤0.

**Remark** **5.** 

*(1)*  *Entropy inequality Equation (97) is satisfied if*
  (98) d(s)0σ¯lk1γilk≥0,
  (99) (s)0m¯lk(s)1γiΘlk≥0,
  (100) $$\begin{array}{c} and\;\frac{{\bar{q}}_{i}{\bar{g}}_{i}}{\bar{\theta }}\leq 0. \end{array}$$
*(2)*  *Inequalities (98) and (99) require that rate of work due to d(s)0σ¯ and (s)0m¯ be positive. The constitutive theory for $\bar{q}$ must satisfy inequality Equation (100).*
*(3)*  *We derive constitutive theories for d(s)0σ¯, (s)0m¯, and $\bar{q}$ using Equations (92), (93), and (96) based on representation theorem [7,11,12,13,14,15,16,17,18,19,20,21,22,23,24,25,26].*

#### 4.7.1. Constitutive Theory for d(s)0σ¯

Consider Equation (92) defining argument tensors of d(s)0σ¯. Let [G~iσ];i=1,2,…,Nε be the combined generators of the argument tensors of d(s)0σ¯ in Equation (92) that are symmetric tensors of rank two, then d(s)0σ¯ in the current configuration can be represented as a linear combination of $\left[I\right]$ and G~iσ;i=1,2,…,Nε:

(101) d(s)0σ¯=α~0σI+∑i=1Nεα~iσG~iσ.

The coefficients α~iσ;i=0,1,…,Nε are functions of $\bar{\rho }$, $\bar{\theta }$, and I~jσ;j=1,2,…,Mε. I~jσ;j=1,2,…,Mε are the combined invariants of the same argument tensors of d(s)0σ¯ as in Equation (92), that is,

(102) α~iσ=α~iσρ¯,I~jσ,θ¯;i=0,1,…,Nεj=1,2,…,Mε.

To determine material coefficients in the constitutive theory Equation (101), we expand α~iσ;i=0,1,…,Nε in Taylor series in I~jσ;j=1,2,…,Mε and $\bar{\theta }$ about a known configuration $\underline{\Omega }$ and retain only up to linear terms in I~jσ;j=1,2,…,Mε and $\bar{\theta }$ (for simplicity):

(103) α~iσ=α~iσΩ_+∑j=1Mε∂α~iσ∂I~jσΩ_I~jσ−I~jσ|Ω_+∂α~iσ∂θ¯Ω_θ¯−θ¯Ω_;i=0,1,…,Nε.

We substitute α~iσ;i=0,1,…,Nε from Equation (103) in Equation (101) and collect coefficients (defined in $\underline{\Omega }$ configuration) of the terms defined in the current configuration and introduce new coefficients for the terms defined in the known configuration, $\underline{\Omega }$. Then, we can obtain the following:

(104) d(s)0σ¯=0σ¯Ω_I+∑j=1Mεσa_jI~jσI+∑i=1Nεσb_iG~iσ+∑i=1Nε∑j=1Mεσc_ijI~jσG~iσ−∑i=1Nεσd_iθ¯−θ¯Ω_G~iσ−σα_tmθ¯−θ¯Ω_I.

In Equation (104) the quantities ${}^{\sigma }{\underline{a}}_{j}$, ${}^{\sigma }{\underline{b}}_{i}$, ${}^{\sigma }{\underline{c}}_{ij}$, ${}^{\sigma }{\underline{d}}_{i}$, and ${}^{\sigma }{\underline{\alpha }}_{\mathrm{tm}}\;;\;i=1,2,\ldots ,N_{\varepsilon }\;\mathrm{and}\;j=1,2,\ldots ,M_{\varepsilon }$ are material coefficients defined in the known configuration $\underline{\Omega }$. The material coefficients are functions of ${\left.\bar{\rho }\right|}_{\underline{\Omega }}$, ${\left.\bar{\theta }\right|}_{\underline{\Omega }}$, and I~jσΩ_;j=1,2,…,Mε. This constitutive theory requires $\left(M_{\varepsilon }+2N_{\varepsilon }+M_{\varepsilon }N_{\varepsilon }+1\right)$ material coefficients.

**Remark** **6.** 

*(1)*  *The constitutive theory Equation (104) is based on integrity, hence utilizes complete basis, but unfortunately requires determination of too many material coefficients. This constitutive theory utilizes convected time derivatives of the strain tensor up to orders $n_{\varepsilon }$.*
*(2)*  *We can derive simplified constitutive theory from Equation (104) by only retaining the desired terms on the right hand side of Equation (104). In the following we consider a constitutive theory that is linear in convected time derivatives of the strain tensor in which higher degree and all product terms are neglected. Introducing more familiar notation for material coefficients:*
  (105) d(s)0σ¯=0σ¯Ω_I+∑i=1nε2μiiγ+∑i=1nελitriγI−σα_tmθ¯−θ¯Ω_I.
*This constitutive theory contains each strain rate and its trace and is obviously nonlinear due to the fact that $\left[{}^{\left(i\right)}\gamma \right]\;;\;i=2,3,\ldots ,n_{\varepsilon }$ are nonlinear functions of the components of the velocity gradient tensor. Each convected time derivative of the strain tensor requires two material coefficients $\left({}^{}\mu_{i},\;{}^{}\lambda_{i}\right)$.*
*(3)*  *The only possible linear constitutive theory for d(s)0σ¯ is given by*
  (106) d(s)0σ¯=0σ¯Ω_I+2μ11γ+λ1tr1γI−σα_tmθ¯−θ¯Ω_I.
*This constitutive theory is basis independent as $\left[\gamma_{\left(1\right)}\right]=\left[\gamma^{\left(1\right)}\right]=\left[\bar{D}\right]=\left[{}^{\left(1\right)}\gamma^{J}\right]$, hence d(s)0σ¯ is basis independent as well and we can write Equation (106) as*
  (107) $$\left[{}_{d}\;\left({}_{s}\bar{\sigma }\right)\right]={\left.{\bar{\sigma }}^{0}\right|}_{\underline{\Omega }}\left[I\right]+2{}^{}\mu_{1}\left[\bar{D}\right]+{}^{}\lambda_{1}\left(tr\left[\bar{D}\right]\right)\left[I\right]-{}^{\sigma }{\underline{\alpha }}_{tm}\left(\bar{\theta }-{\left.\bar{\theta }\right|}_{\underline{\Omega }}\right)\left[I\right].$$
*In which dsσ¯=dsσ¯0=dsσ¯0=d(s)0σ¯J as right hand side of Equation (107) is basis independent.*
*(4)*  *It is instructive to consider constitutive theory with $n_{\varepsilon }=1$, in which case we have*
  (108) d(s)0σ¯=d(s)0σ¯ρ¯,1γ,θ¯.
*Hence, in this case we have $\left[I\right],\;\left[{}^{\left(1\right)}\gamma \right]=\left[\bar{D}\right],$ and ${\left[{}^{\left(1\right)}\gamma \right]}^{2}={\left[\bar{D}\right]}^{2}$ as combined generators of the argument tensors of d(s)0σ¯:*
  (109) d(s)0σ¯=α~0σI+α~1σ1γ+α~2σ1γ2,
  (110) ord(s)0σ¯=α~0σI+α~1σD¯+α~2σD¯2
  (111) inwhichα~iσ=α~iσρ¯,θ¯,ID¯,IID¯,IIID¯;i=0,1,2.
  (112) Ifwechooseiγ=G~1σ,iγ2=G~2σ;i.e.Nε=2andI~1σ=ID¯,I~2σ=IID¯,andI~3σ=IIID¯;Mε=3
*then the resulting constitutive theory is given by Equation (104) with $N_{\varepsilon }=2$, and $M_{\varepsilon }=3$. The generators and the invariants are defined in Equation (112).*

#### 4.7.2. Constitutive Theory for (s)0m¯

We consider Equation (93) defining argument tensors of (s)0m¯. Let G~im;i=1,2,…,Nm be the combined generators of the argument tensors of rank two, then (s)0m¯ can be represented by a linear combination of $\left[I\right]$ and G~im;i=1,2,…,Nm:

(113) (s)0m¯=α~0mI+∑i=1Nmα~imG~im.

The coefficients α~im;i=0,1,…,Nm are functions of $\bar{\rho }$, $\bar{\theta }$, and I~jm;j=1,2,…,Mm. I~jm;j=1,2,…,Mm are the combined invariants of the same argument tensors of (s)0m¯ as in (93), that is,

(114) α~im=α~imρ¯,I~jm,θ¯;j=1,2,…,Mm.

To determine material coefficients in the constitutive theory Equation (113), we expand α~im;i=0,1,…,Nm in Taylor series in I~jm;j=1,2,…,Mm and $\bar{\theta }$ about a known configuration $\underline{\Omega }$ and retain only up to linear terms in I~jm and $\bar{\theta }$ (for simplicity):

(115) α~im=α~imΩ_+∑j=1Mm∂α~im∂I~jmΩ_I~jm−I~jmΩ_+∂α~im∂θ¯Ω_θ¯−θ¯Ω_;i=0,1,…,Nm.

We substitute α~im;i=0,1,…,Nm in Equation (113) and collect coefficients (those defined in $\underline{\Omega }$ configuration) of the terms defined in the current configuration and introduce new coefficients for the terms defined in the known configuration $\underline{\Omega }$. Then, we obtain the following:

(116) (s)0m¯=0m¯Ω_I+∑j=1Mmma_jI~jmI+∑i=1Nmmb_iG~im+∑i=1Nm∑j=1Mmmc_ijI~jmG~im−∑i=1Nmmd_iθ¯−θ¯Ω_−mα_tmθ¯−θ¯Ω_I.

In Equation (116), ${}^{m}{\underline{a}}_{j}$, ${}^{m}{\underline{b}}_{i}$, ${}^{m}{\underline{c}}_{ij}$, ${}^{m}{\underline{d}}_{i}$, and ${}^{m}{\underline{\alpha }}_{\mathrm{tm}}\;;\;i=1,2,\ldots ,N_{m}\;;\;j=1,2,\ldots ,M_{m}$ are material coefficients defined in the known configuration $\underline{\Omega }$. The material coefficients are functions of ${\left.\bar{\rho }\right|}_{\underline{\Omega }}$, ${\left.\bar{\theta }\right|}_{\underline{\Omega }}$, and I~jmΩ_;j=1,2,…,Mm. This constitutive theory requires $\left(M_{m}+2N_{m}+N_{m}M_{m}+1\right)$ material coefficients.

**Remark** **7.** 

*(1)*  *The constitutive theory Equation (116) is based on integrity, hence utilizes complete basis, but unfortunately requires determination of too many material coefficients. This constitutive theory utilizes symmetric part of the gradients of the convected rotation rates up to orders $n_{\Theta }$.*
*(2)*  *It is possible to derive simplified constitutive theory for (s)0m¯ using Equation (116) by retaining only the desired terms on the right side of Equation (116). In the following we consider a constitutive theory for (s)0m¯ that is linear in the (s)iγiΘ;i=1,2,…,nΘ in which the higher degree terms of (s)iγiΘ as well as all product terms are neglected:*
  (117) (s)0m¯=0m¯Ω_I+∑i=1nΘ2Θμi(s)iγiΘ+∑i=1nΘΘλitr(s)iγiΘI−mα_tmθ¯−θ¯Ω_I.
*This constitutive theory contains (s)iγiΘ and tr(s)iγiΘ,i=1,2,…,nΘ and is obviously nonlinear due to the fact that (s)iγiΘ;i=2,3,…,nΘ are nonlinear functions of the components of the velocity gradient tensor. Consideration of each rate requires two material coefficients ${}^{\Theta }\mu_{i}$ and ${}^{\Theta }\lambda_{i}$.*
*(3)*  *The only possible linear constitutive theory for (s)0m¯ is given by:*
  (118) (s)0m¯=0m¯Ω_I+2Θμ1(s)iγiΘ+Θλ1tr(s)iγiΘI−mα_tmθ¯−θ¯Ω_I
*in which tr(s)iγiΘ=0, hence in this constitutive theory only one material coefficient is associated with the rate of order one.*
*(4)*  *It is instructive to consider a constitutive theory for (s)0m¯ with $n_{\Theta }=1$, in which case we have*
  (119) (s)0m¯=(s)0m¯ρ¯,(s)1γiΘ,θ¯.
  *Hence,*
  (120) (s)0m¯=α~0mI+α~1m(s)1γiΘ+α~2m(s)1γiΘ2
  (121) inwhichα~im=α~imρ¯,θ¯,I(s)1γiΘ,II(s)1γiΘ,III(s)1γiΘ;i=0,1,2
  (122) Ifwechoose(s)1γiΘ=G~1m,(s)1γiΘ2=G~2m;Nm=2andI~1m=I(s)1γiΘ,I~2m=II(s)1γiΘ,I~3m=III(s)1γiΘ
*then the resulting constitutive theory is given by Equation (116) with $N_{m}=2,\;M_{m}=3$. The generators and invariants are defined in Equation (122).*

#### 4.7.3. Constitutive Theory for $\bar{q}$

Consider the argument tensors of $\bar{q}$ in Equation (96), that is,

(123) $$\bar{q}=\bar{q}\;\left(\bar{\rho },\;\bar{g},\;\bar{\theta }\right).$$

Using representation theorem (following Reference [7]) we can derive the following constitutive theory, keeping in mind that here the generator is $\bar{g}$ and the invariant is $\bar{g}\cdot \bar{g}$:

(124) $$\bar{q}=-k\bar{g}-k_{1}\left(\bar{g}\cdot \bar{g}-{\left.\left(\bar{g}\cdot \bar{g}\right)\right|}_{\underline{\Omega }}\right)\bar{g}-k_{2}\left(\bar{\theta }-{\left.\bar{\theta }\right|}_{\underline{\Omega }}\right)\bar{g},$$

in which *k*, $k_{1}$, and $k_{2}$ can be functions of $\bar{\rho }$, ${\left.\bar{\theta }\right|}_{\underline{\Omega }}$, and ${\left.\left(\bar{g}\cdot \bar{g}\right)\right|}_{\underline{\Omega }}$. A linear constitutive theory for $\bar{q}$ is given by

(125) $$\bar{q}=-k\bar{g}.$$

This is the Fourier heat conduction law.

## 5. Complete Mathematical Model

The equations resulting from the conservation and balance laws and constitutive theories, based on integrity and simplified are presented in this section. It is shown that this mathematical model has closure.

(126) $$\begin{array}{c} \frac{\partial \bar{\rho }}{\partial t}+\frac{\partial }{\partial {\bar{x}}_{i}}\left(\bar{\rho }{\bar{v}}_{i}\right)=0\;\left(\mathrm{CM}\right), \end{array}$$

(127) $$\begin{array}{c} \bar{\rho }\frac{\partial {\bar{v}}_{i}}{\partial t}+\bar{\rho }{\bar{v}}_{j}\frac{\partial {\bar{v}}_{i}}{\partial {\bar{x}}_{j}}-\bar{\rho }{\bar{F}}_{i}^{b}-\frac{\partial }{\partial {\bar{x}}_{j}}\left({}^{\left(0\right)}{\bar{\sigma }}_{ji}\right)=0\;\left(\mathrm{BLM}\right), \end{array}$$

(128) $$\begin{array}{c} {}^{\left(0\right)}{\bar{m}}_{pk,p}+\epsilon_{ijk}{}^{\left(0\right)}{\bar{\sigma }}_{ij}=0\;\left(\mathrm{BAM}\right), \end{array}$$

(129) $$\begin{array}{c} \epsilon_{ijk}{}^{\left(0\right)}{\bar{m}}_{ij}=0\;\left(\mathrm{BMM}\right), \end{array}$$

(130) ρ¯De¯Dt+∂q¯i∂x¯i−(s)0σ¯jiD¯ij−(s)0m¯ji(s)1γiΘij=0FLT,

(131) q¯ig¯iθ¯−d(s)0σ¯lk1γilk−(s)0m¯lk(s)1γiΘlk≤0SLT.

Constitutive theories for e(s)0σ¯:

(132) e(s)0σ¯=p¯θ¯I;Incompressible

(133) e(s)0σ¯=p¯ρ¯,θ¯I;Compressible

Constitutive theories for d(s)0σ¯, (s)0m¯, and $\bar{q}$:

(a) Based on integrity:
  (134) d(s)0σ¯=0σ¯Ω_I+∑j=1Mεσa_jI~jσI+∑i=1Nεσb_iG~iσ+∑i=1Nε∑j=1Mεσc_ijI~jσG~iσ−∑i=1Nεσd_iθ¯−θ¯Ω_G~iσ−σα_tmθ¯−θ¯Ω_I,
  (135) (s)0m¯=0m¯Ω_I+∑j=1Mmma_jI~jmI+∑i=1Nmmb_iG~im+∑i=1Nm∑j=1Mmmc_ijI~jmG~im−∑i=1Nmmd_iθ¯−θ¯Ω_−mα_tmθ¯−θ¯Ω_I,
  (136) $$\bar{q}=-k\bar{g}-k_{1}\left(\bar{g}\cdot \bar{g}-{\left.\left(\bar{g}\cdot \bar{g}\right)\right|}_{\underline{\Omega }}\right)\bar{g}-k_{2}\left(\bar{\theta }-{\left.\bar{\theta }\right|}_{\underline{\Omega }}\right)\bar{g}.$$
(b) Simplified theories (neglecting product terms):
  (137) d(s)0σ¯=0σ¯Ω_I+∑i=1nε2μiiγ+∑i=1nελitriγI−σα_tmθ¯−θ¯Ω_I,
  (138) (s)0m¯=0m¯Ω_I+∑i=1nΘ2Θμi(s)iγiΘ+∑i=1nΘΘλitr(s)iγiΘI−mα_tmθ¯−θ¯Ω_I,
  (139) $$\bar{q}=-k\bar{g}.$$

This mathematical model consists of conservation of mass (1), balance of linear momenta (3), balance of angular momenta (3), first law of thermodynamics (1), constitutive theories: d(s)0σ¯(6), (s)0m¯(6), $\bar{q}\;\left(3\right)$, a total of twenty-three equations in the dependent variables: $\bar{\rho }\;\left(1\right)$, $\bar{v}\;\left(3\right)$, ${}^{\left(0\right)}\bar{\sigma }\;\left(9\right)$, (s)0m¯(6), $\bar{q}\;\left(3\right)$, $\bar{\theta }\;\left(1\right)$, a total of twenty three dependent variables, hence this mathematical model has closure. We note that $\bar{e}=\bar{e}\;\left(\bar{\rho },\;\bar{\theta }\right)$, and $\bar{p}=\bar{p}\;\left(\bar{\rho },\;\bar{\theta }\right)$ or $\bar{p}=\bar{p}\;\left(\bar{\theta }\right)$, hence $\bar{e}$ and $\bar{p}$ are not dependent variables in the mathematical model.

## 6. Summary and Conclusions

In this section, we present a summary of the work presented in the paper, highlight new and meritorious features and present some conclusions.

(1) Role of entropy inequality in the derivation of the constitutive theories for Cauchy stress tensor, Cauchy moment tensor, and heat vector are discussed in Section 3, remark 2.
(2) The paper considers the conservation and balance laws of non-classical mechanics [1,2,3] in Eulerian description in which internal rotation rates naturally arise due to anti-symmetric part of the velocity gradient tensor.
(3) Surana et al. [6] have shown that in this NCCM, the conservation and balance laws of CCM need modifications and a new balance law ‘balance of moment of moments’ is essential to ensure dynamic equilibrium of the deforming fluent continua.
(4) It has been shown [3] that, in this physics, a more complete constitutive theory for the symmetric part of the Cauchy stress tensor can be derived using convected time derivatives of the Green’s or Almansi strain tensor. However, the constitutive theories for the symmetric Cauchy moment tensor have been limited to the symmetric part of the gradients of rotation rates.
(5) In this paper, we show that parallel to the convected time derivatives of the strain tensors (up to orders $n_{\varepsilon }$), it is also possible to establish symmetric gradient tensors of the higher order rotation rates. The derivation presented in this paper derives a recursive form through which rotation rates of any order ($n_{\Theta }$) can be obtained.
(6) The constitutive theories for Cauchy stress and Cauchy moment tensors are derived by:
  *(i)*  first establishing their argument tensors using conjugate pairs in the entropy inequality;
  *(ii)*  then augmenting these by the higher order convected time derivatives;
  *(iii)*  consideration of the argument tensors of $\bar{\phi }$ using principle of equipresence and using $\overset{\;.}{\bar{\phi }}$ in the entropy inequality;
  *(iv)*  decompositions of Cauchy stress and Cauchy moment tensors into equilibrium and deviatoric parts.
(7) Constitutive theory for equilibrium stress tensor is established for compressible and incompressible fluent continua.
(8) Constitutive theories for deviatoric part of the symmetric Cauchy stress and Cauchy moment tensor and heat vector are derived using representation theorem. These theories are based on integrity (complete basis), hence are complete and are naturally nonlinear in terms of their argument tensors. Simplified constitutive theories are also presented.
(9) The dissipation mechanism in such non-classical fluent continua is a higher order rate mechanism: arising from the strain rates (up to orders $n_{\varepsilon })$ as well as the symmetric gradients of the rotation rates (up to orders $n_{\Theta }$). We note that $n_{\varepsilon }=1$ and $n_{\Theta }=0$ (CCM) gives rise to standard constitutive theory for Cauchy stress tensor used for Newtonian fluids (compressible as well as incompressible).
(10) Model problem studies considering $n_{\varepsilon }\geq 1$ and $n_{\Theta }\geq 1$ will be presented in a forthcoming paper.

## Abbreviations

The following abbreviations are used in this manuscript:

BAM	Balance of angular momenta
BLM	Balance of linear momenta
BMM	Balance of moment of moments
CM	Conservation of mass
CCM	Classical continuum mechanics
FLT	First law of thermodynamics
NCCM	Non-classical continuum mechanics
SLT	Second law of thermodynamics
